# Depressive symptoms mediate the association between sleep disorders and gallstone disease: a causal mediation analysis of NHANES 2017–2020

**DOI:** 10.3389/fpsyt.2025.1434889

**Published:** 2025-04-17

**Authors:** Yisen Hou, Rui Li, Zhen Xu, Wenhao Chen, Zhiwen Li, Weirong Jiang, Yong Meng, Jianli Han

**Affiliations:** ^1^ Department of Oncology Surgery, Xi’an No.3 Hospital, the Affiliated Hospital of Northwest University, Xi’an, Shanxi, China; ^2^ Department of General Surgery, Shanxi Bethune Hospital, Shanxi Academy of Medical Sciences, Third Hospital of Shanxi Medical University, Taiyuan, Shanxi, China

**Keywords:** gallstones, sleep disorders, depression, national health and nutrition examination survey, causal mediation analysis

## Abstract

**Background:**

Gallstones are a prevalent digestive system disorder with significant health implications. Recent research suggests that sleep disorders, such as insomnia and obstructive sleep apnea hypopnea syndrome, may influence the development of gallstones through various metabolic pathways. Depression, often accompanying sleep disorders, may play a mediating role in this relationship. This study uses data from the 2017–2020 National Health and Nutrition Examination Survey (NHANES) to explore the potential mediating role of depression in the association between sleep disorders and gallstones.

**Methods:**

We analyzed data from 7,868 adults aged 20 and older from NHANES 2017–2020. Gallstones were defined based on self-reported medical diagnoses. Sleep disorders were assessed through self-reported sleep difficulties, and depressive symptoms were measured using the Patient Health Questionnaire-9 (PHQ-9) scale. Logistic regression models evaluated direct associations between sleep disorders, depressive symptoms, and gallstones. Causal mediation analysis further examined the mediating role of depressive symptoms. Finally, subgroup analyses were performed by age, sex, and obesity status.

**Results:**

Both sleep disorders (OR = 2.00; 95% CI, 1.73-2.32; P<0.001) and depressive symptoms (OR = 2.09; 95% CI, 1.70-2.56; P<0.001) were significantly associated with gallstones, with results remaining significant after adjusting for confounders. A significant association was also observed between sleep disorders and depressive symptoms (OR = 5.53; 95% CI, 4.71-6.50; P<0.001). Mediation analysis indicated that depressive symptoms partially mediate the relationship between sleep disorders and gallstones, with an average causal mediation effect (ACME) of 0.00720 (95% CI, 0.00299-0.01220; P<0.001) and an average direct effect (ADE) of 0.0305 (95% CI, 0.0129-0.0488; P<0.001). Depression mediates 18.89% (95% CI, 0.0704-0.4096; P<0.001) of the association between sleep disorders and gallstones. Subgroup analyses showed significant mediation by depressive symptoms in individuals aged 40-59, males, and both obese and non-obese groups (all P<0.05), although no significant mediation was found in females (P>0.05).

**Conclusion:**

This study demonstrates a significant association between sleep disorders and gallstones, with depressive symptoms playing a partial mediating role. Improving depressive symptoms may help reduce the risk of gallstones associated with sleep disorders.

## 1 Introduction

Gallstone disease is a common digestive system disorder, affecting approximately 10% to 20% of adults worldwide, with incidence rates varying by region and population (1). Higher prevalence is observed in Western countries, particularly among women, individuals with obesity, and the elderly (2, 3). The clinical presentation of gallstone ranges from asymptomatic “silent” stones to severe complications such as cholecystitis, pancreatitis, and even an increased risk of cholangiocarcinoma (4, 5). The formation of gallstones is known to be influenced by multiple factors, including age, sex, obesity, metabolic syndrome, dietary habits, genetic predisposition, pregnancy, diabetes, and biliary infections (1, 6, 7). Recently, however, research has begun to explore potential risk factors beyond the traditional ones, particularly those related to metabolism and mental health, such as sleep disorders (8, 9).

Sleep disorders, especially insomnia and obstructive sleep apnea hypopnea syndrome (OSAHS), represent another widespread public health issue affecting the quality of life of millions of people (10). Numerous studies have indicated that sleep disorders are closely linked to various metabolic diseases (e.g., obesity, diabetes), which are known risk factors for gallstone formation (11, 12). Therefore, sleep disorders may influence gallstone formation through multiple metabolic pathways, including insulin resistance, dysregulated lipid metabolism, and weight gain (13–15).

Furthermore, there is a bidirectional relationship between depression and sleep disorders: sleep disorders are both a risk factor for depression and a typical symptom of it (16–18). The relationship between depression and metabolic diseases such as obesity and metabolic syndrome is well-documented, and these metabolic conditions are closely related to gallstone formation (19–21). Thus, depressive symptoms might mediate the relationship between sleep disorders and gallstones by exacerbating metabolic abnormalities and increasing chronic inflammation. However, research on the mediating role of depressive symptoms in the relationship between sleep disorders and gallstones remains limited, with no definitive reports to date.

Therefore, this study aims to use data from the 2017–2020 National Health and Nutrition Examination Survey (NHANES) to systematically explore the potential association between sleep disorders and gallstones, with a focus on evaluating the potential mediating role of depressive symptoms. This study addresses this gap by examining whether depressive symptoms mediate the association between sleep disorders and gallstone disease, offering novel insights into its multifactorial etiology. The specific research questions include: (1) Is there a significant association between sleep disorders and gallstones? (2) Do depressive symptoms mediate this association?

## 2 Materials and methods

### 2.1 Study design and population

We utilized data from the 2017-2020 NHANES to investigate the relationship between sleep disorders and gallstone risk, and to assess the mediating role of depressive symptoms. NHANES data is maintained by the Centers for Disease Control and Prevention (CDC) and is updated biennially. This study combined data from 2017-2020, and due to the lack of national representativeness for the 2019-2020 data, it was merged with the 2017-2018 cycle data for analysis. More information about the NHANES program can be found on the CDC website.

**Figure 1:**
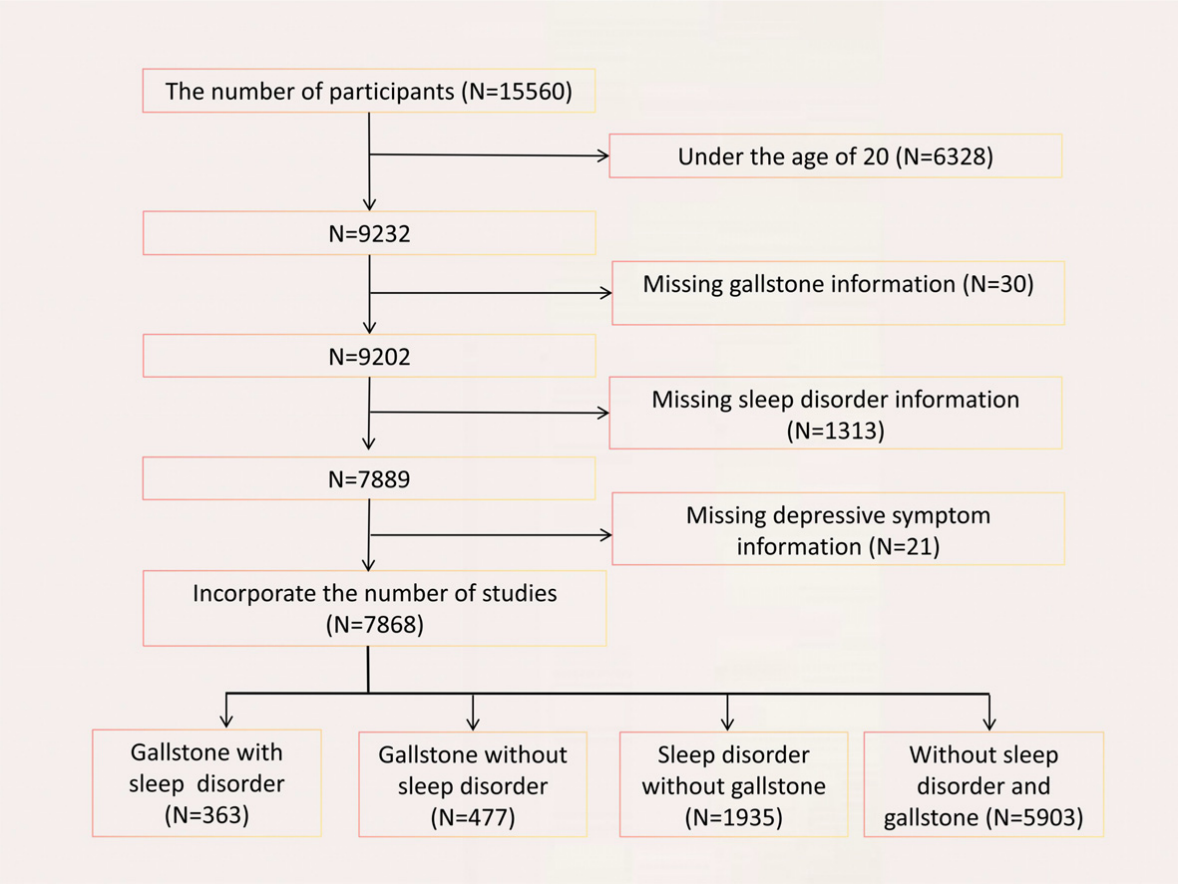
Flowchart of participant selection. A total of 15560 participants were included, and after exclusions, the final number of participan was 7868.

Participants were included based on the following eligibility criteria: 1) age ≥ 20 years; 2) complete data on sleep disorders, depressive symptoms, and gallstones (18). After rigorous screening, a final cohort of 7,868 eligible participants was identified. (**Figure 1**)

### 2.2 Definition of gallstones

Information on gallstones was obtained from self-reported responses to the question: “MCQ550—Has a doctor ever said you have gallstones?” A response indicating a diagnosis was considered as having gallstones, while a negative response was considered as not having gallstones.

### 2.3 Definition of sleep disorders

Data on sleep disorders were based on self-reported responses to the question: “SLQ050 - Ever told doctor had trouble sleeping?” Respondents who answered “Yes” were classified as having sleep disorders, while those who answered “No” were classified as not having sleep problems.

### 2.4 Definition of depressive symptoms

Depressive symptoms were assessed using the PHQ-9 scale, which is widely recognized as a reliable and accurate screening tool for depression (22). The PHQ-9 total score ranges from 0 to 27, with higher scores indicating more severe depression. A PHQ-9 score of ≥10 was considered indicative of depressive symptoms, while a score of <10 was considered indicative of no depressive symptoms. Detailed information about the PHQ-9 scale can be found in the NHANES database.

### 2.5 Definition of covariates

Covariates included in this study were: age (categorized as 20-39 years, 40-59 years, ≥60 years), sex, race, marital status, education level, BMI (with obesity defined as BMI ≥30), smoking status (never, former, current), alcohol consumption (based on 24-hour dietary recall), physical activity (based on the PAQ questionnaire), medication use, and self-reported hypertension, hyperlipidemia, and diabetes status. Specific definitions of some covariates are detailed in **Table 1**. These variables were selected based on a review of existing literature and their potential impact on the relationship between sleep disorders and gallstones.

**Table 1:** Definitions of covariates.

Variable Name	Definition and Classification
Age	20-39 years, 40-59 years, ≥60 years
Sex	Male, Female
Race	Non-Hispanic White, Non-Hispanic Black, Mexican American, Other Race
Marital Status	Married/Co-habiting, Single/Widowed/Divorced
Education Level	< High school, Completed high School,> High School
BMI	Calculated as weight (kg) divided by height (m) squared; obesity defined as BMI ≥30
Smoking	Never Smoker (<100 cigarettes), Former Smoker (≥100 cigarettes but not currently smoking), Current Smoker (≥100 cigarettes and currently smoking daily or occasionally)
Alcohol	Drinkers: reported drinking at least once in 24-hour dietary recall; Non-drinkers: no alcohol reported
Physical activity	Sedentary, Moderate Activity, Vigorous Activity, Both moderate and vigorous Activity (based on PAQ questionnaire, including high-intensity activities like running and moderate activities like brisk walking)
Hypertension (23)	Systolic BP ≥130 mmHg or Diastolic BP ≥80 mmHg, or use of antihypertensive medication
Diabetes (24)	Fasting glucose ≥126 mg/dL or HbA1c ≥6.5%, or use of insulin or oral hypoglycemic agents
Hyperlipidaemia (25)	Total Cholesterol (TC) >200 mg/dL, Triglycerides (TG) >150 mg/dL, LDL ≥130 mg/dL, HDL <40 mg/dL (male) or <50 mg/dL (female)
Sleep-related medication use	Use of barbiturates, benzodiazepines, anti-anxiety medications, sedatives, hypnotics; categorized as use or non-use

### 2.6 Statistical analysis

First, descriptive statistics of participants’ baseline characteristics were performed. Continuous variables were described using weighted means and standard errors, while categorical variables were presented with observed frequencies and weighted percentages. For normally distributed variables (based on Shapiro-Wilk test results), one-way ANOVA was used for group comparisons; for non-normally distributed variables, Kruskal-Wallis H test was employed. Comparisons of categorical variables were conducted using chi-square tests or Fisher’s exact tests when applicable.

Next, multivariable logistic regression analysis was used to assess the relationship between depressive symptoms, sleep disorders, and gallstones, adjusting for confounders such as age, sex, race, BMI, smoking status, alcohol consumption, physical activity, hypertension, and diabetes. Sensitivity analysis was conducted to evaluate the impact of sleep-related medication on the outcomes.

To further explore the mediating role of depressive symptoms between sleep disorders and gallstone risk, causal mediation analysis was performed. The causal mediation analysis (using Baron and Kenny’s method combined with bootstrap) decomposed the total effect into direct and indirect effects via the mediator. We employed 1,000 bootstrap resamples to enhance the robustness of estimates and reported the average causal mediation effect (ACME), average direct effect (ADE), and total effect. Additionally, the size of the indirect path effect, proportion of mediation effect, and statistical significance (p-value) were presented.

Furthermore, we conducted an item-level analysis of the PHQ-9 scale to examine the causal mediation effects of individual depressive symptoms (scored as 0 or ≥1) on the relationship between sleep disorders and gallstones. Each item score of 0 indicated no symptom, and ≥1 indicated the presence of symptoms, to evaluate the mediation effect of each symptom.

Finally, subgroup analyses were performed to assess the impact of different age groups (20-39 years, 40-59 years, ≥60 years), sex, and obesity status (BMI ≥30) on gallstone risk, and further mediation analysis was conducted for statistically significant subgroups to evaluate whether depressive symptoms mediate the relationship between sleep disorders and gallstone risk within each subgroup.

## 3 Results

### 3.1 General characteristics of the study population

Baseline demographic characteristics are summarized in **Table 2**. The study population (n = 7,868) was divided into four groups:”Gallstones with Sleep Disorders,”“Gallstones without Sleep Disorders,”“Sleep Disorders without Gallstones,” and “Neither Gallstones nor Sleep Disorders.” The “Gallstones with Sleep Disorders” group had a mean age of 58.06 years, with 11.3% in the 20-39 age group, 38.0% in the 40-59 age group, and 74.7% female. The mean BMI for this group was 34.65 kg/m².

**Table 2:** Characteristics of study participants.

Characteristic	Gallstone with sleep disturbance	Gallstone without sleep disturbance	Sleep disturbance without gallstone	Without sleep disturbance and gallstone	P-value
N	363	477	1935	5093	
Age (Year)	58.06 (14.49)	58.46 (16.55)	53.20 (16.13)	48.54 (17.69)	<0.001
20-39	41 (11.3%)	86 (18.0%)	440 (22.7%)	1836 (36.0%)	
40-59	138 (38.0%)	137 (28.7%)	725 (37.5%)	1616 (31.7%)	
>60	184 (50.7%)	254 (53.2%)	770 (39.8)	1641 (32.2%)	
Gender (%)					<0.001
Male	92 (25.3%)	145 (30.4%)	913 (47.2%)	2711 (53.2%)	
Female	271 (74.7%)	332 (69.6%)	1022 (52.8%)	2382 (46.8%)	
Race (%)					<0.001
Mexican American	37 (10.2%)	71 (14.9%)	166 (8.6%)	640 (12.6%)	
Non-Hispanic White	170 (46.8%)	196 (41.1%)	824 (42.6%)	1599 (31.4%)	
Non-Hispanic Black	74 (20.4%)	96 (20.1%)	497 (25.7%)	1424 (28.0%)	
Other Race	82 (22.6%)	114 (23.9%)	448 (23.2%)	1430 (28.1%)	
Married/live with partner (%)					<0.001
Yes	206 (56.7%)	114 (23.9%)	1031 (53.4%)	3042 (59.8%)	
No	157 (43.3%)	286 (60.0%)	901 (46.6%)	2047 (40.2%)	
Education level (%)					0.162
< High school	23 (6.3%)	26 (5.5%)	113 (5.8%)	384 (7.5%)	
Completed high School	40 (11.0%)	58 (12.2%)	210 (10.9%)	537 (10.6%)	
> High School	300 (82.6%)	393 (82.4%)	1611 (83.3%)	4167 (81.9%)	
Poverty income ratio	2.45 (1.63)	2.62 (1.51)	2.63 (1.65)	2.64 (1.63)	0.273
BMI (kg/m^2^)	34.65 (9.23)	32.06 (8.01)	31.32 (8.15)	29.09 (6.89)	<0.001
Obesity	235 (66.0%)	272 (58.6%)	972 (88.6%)	1888 (37.4%)	
Non-Obesity	121 (34.0%)	192 (41.4%)	939 (49.1%)	3160 (62.6%)	
Waist circumference (cm)	111.12 (18.12)	107.03 (16.58)	104.44 (17.68)	98.69 (16.38)	<0.001
Smoking (%)					<0.001
Never	175 (48.3%)	272 (57.1%)	961 (49.7%)	3132 (61.5%)	
Former	112 (30.9%)	143 (30.0%)	548 (28.3%)	1082 (21.2%)	
Current	75 (20.7%)	61 (12.8%)	426 (22.0%)	878 (17.2%)	
Alcohol (%)					0.001
Yes	27 (7.9%)	23 (5.3%)	206 (11.4%)	449 (9.8%)	
No	316 (92.1%)	414 (94.7%)	1595 (88.6%)	4149 (90.2%)	
Physical activity (%)					0.001
Inactive	187 (51.5%)	275 (57.7%)	941 (48.6%)	2643 (51.9%)	
Moderate	103 (28.4%)	106 (22.2%)	462 (23.9%)	1124 (24.1%)	
Vigorous	10 (2.8%)	14 (2.9%)	99 (5.1%)	214 (4.2%)	
Both moderate and vigorous	63 (17.4%)	82 (17.2%)	433 (22.4%)	1112 (21.8%)	
PHQ-9	6.40 (5.32)	3.12 (4.28)	5.43 (5.20)	2.27 (3.28)	<0.001
Depress (%)					<0.001
Yes	96 (26.4%)	39 (8.2%)	378 (19.5%)	211 (4.1%)	
No	267 (73.6%)	438 (91.8%)	1557 (80.5%)	4882 (95.9%)	
Diabetes (%)					<0.001
Yes	143 (39.4%)	137 (28.7%)	466 (24.1%)	867 (17.0%)	
No	220 (60.6%)	340 (71.3%)	1469 (75.9%)	4226 (83.0%)	
Hypertension (%)					<0.001
Yes	266 (73.3%)	309 (64.8%)	1316 (68.0%)	2606 (51.2%)	
No	97 (26.7%)	168 (35.2%)	619 (32.0%)	2487 (48.8%)	
Hyperlipidaemia (%)					<0.001
Yes	296 (84.6%)	369 (79.5%)	1466 (78.4%)	3310 (68.6%)	
No	54 (15.4%)	95 (20.5%)	404 (21.6%)	1518 (31.4%)	
Sleep-related medication (%)					<0.001
Yes	18 (5.0%)	2 (0.4%)	34 (1.8%)	16 (0.3%)	
No	345 (95.0%)	475 (99.6%)	1901 (98.2%)	5077 (99.7%)	

In comparisons of depressive and sleep disorder groupings, significant differences in PHQ-9 scores were observed among the “Depression with Sleep Disorders,” “Sleep Disorders without Depression,” “Depression without Sleep Disorders,” and “Neither Depression nor Sleep Disorders” groups (mean scores of 6.40 ± 5.32, 3.12 ± 4.28, 5.43 ± 5.20, and 2.27 ± 3.28, respectively; p < 0.001). Participants with gallstones and/or sleep disorders exhibited higher PHQ-9 scores and greater prevalence of depressive symptoms compared to those without either condition.

### 3.2 Relationships between sleep disorders, depressive symptoms, and gallstones

**Table 3** presents the relationships between depressive symptoms, sleep disorders, and gallstones, as well as the association between sleep disorders and depressive symptoms. When gallstones were considered as the outcome variable, the analysis revealed that sleep disorders (OR = 2.00; 95% CI, 1.73-2.32; P<0.001) and depressive symptoms (OR = 2.09; 95% CI, 1.70-2.56; P<0.001) were significantly associated with a higher prevalence of gallstones. After adjusting for age, gender, race/ethnicity, education level, marital status, smoking, alcohol consumption, physical activity, BMI, diabetes status, dyslipidemia status, hypertension status, and use of sleep-related medications, sleep disorders (OR = 1.49; 95% CI, 1.26-1.76; P<0.001) and depressive symptoms (OR = 1.77; 95% CI, 1.39-2.24; P<0.001) remained significantly associated with a higher prevalence of gallstones.

**Table 3:** Association between depressive symptoms, sleep disorders, and gallstone among adults over 20 years of age in 2017–2020 NHANES.

Outcomes	Gallstone OR (95%CI) P value		Depressive symptoms OR(95%CI) P value	
Variables	Model 1	Model 2	Model 1	Model 2
Sleep disorders	2.00(1.73,2.32) <0.001	1.49(1.26,1.76) <0.001	5.53(4.71,6.50) <0.001	5.45(4.53,6.56) <0.001
Depressive symptoms	2.09(1.70,2.56) <0.001	1.77(1.39,2.24) <0.001	–	–

When depressive symptoms were considered as the outcome variable, sleep disorders (OR = 5.53; 95% CI, 4.71-6.50; P<0.001) were significantly associated with a higher prevalence of depressive symptoms. Even after adjusting for confounding factors, sleep disorders (OR = 5.45; 95% CI, 4.53-6.56; P<0.001) remained significantly associated with a higher prevalence of depressive symptoms.

Additionally, we further investigated the relationships between sleep disorders, depressive symptoms, and gallstones among participants not using sleep-related medications (see **Supplementary Table 1**). In this subset of the population, sleep disorders (OR = 1.94; 95% CI, 1.67-2.25; P<0.001) and depressive symptoms (OR = 2.09; 95% CI, 1.68-2.54; P<0.001) were significantly associated with a higher prevalence of gallstones. Sleep disorders (OR = 5.37; 95% CI, 4.56-6.33; P<0.001) were also significantly associated with a higher prevalence of depressive symptoms. These relationships remained significant after adjusting for confounding factors.

### 3.3 Causal mediation analysis

**Figure 2:**
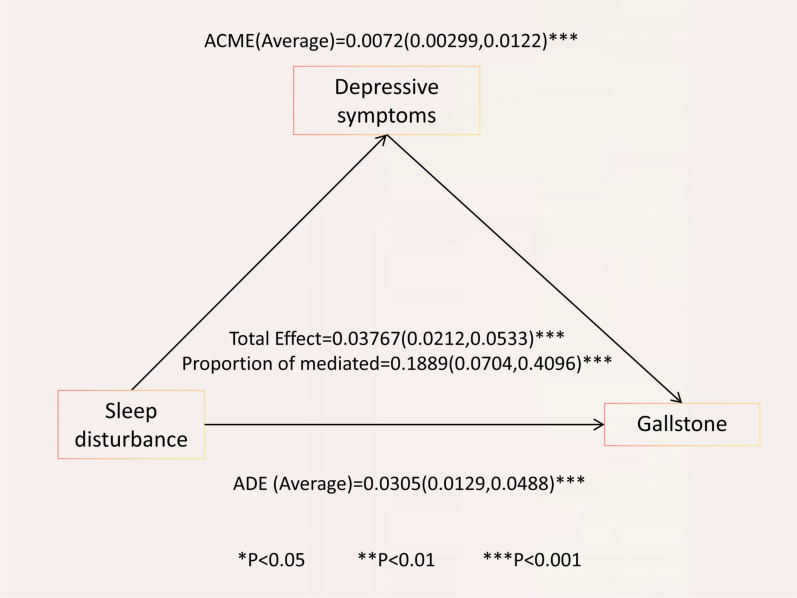
Causal mediation analysis of depressive symptoms in the relationship between sleep disorders and gallstone among adults over 20 years of age in 2017–2020 NHANES. ACME, average causal mediation effect; ADE, average direct effect; Proportion mediated = ACME (Average)/total effect.

**Table 4** and **Figure 2** present the detailed results of the causal mediation analysis. The results indicate that both direct and indirect effects play significant roles in increasing the likelihood of gallstones resulting from sleep disorders. The total effect was 0.03767 (95% CI, 0.0212-0.0533; P < 0.001), with the average causal mediation effect (ACME) being 0.00720 (95% CI, 0.00299-0.01220; P < 0.001) and the average direct effect (ADE) being 0.0305 (95% CI, 0.0129-0.0488; P < 0.001). The proportion of the effect mediated was 0.1889 (95% CI, 0.0704-0.4096; P < 0.001). When confounding factors were not adjusted, the total effect was 0.0725 (95% CI, 0.0569-0.0902; P < 0.001), with the proportion of the mediated effect being 0.13348 (95% CI, 0.0715-0.2228; P < 0.001).

**Table 4:** Causal mediation analysis of depressive symptoms in the association between sleep disorders and gallstones among adults over 20 years of age in 2017–2020 NHANES.

Type	Estimate (95% CI)	P value	Estimate (95% CI)	P value
	Model 1		Model 2	
ACME (control)	0.00773 (0.00399,0.01250)	<0.001	0.00650 (0.00259,0.01119)	<0.001
ACME (treated)	0.01184 (0.00629,0.01821)	<0.001	0.00790 (0.00336,0.01314)	<0.001
ADE (control)	0.06064 (0.0445,0.0782)	<0.001	0.02977 (0.0124,0.0481)	<0.001
ADE (treated)	0.06475 (0.0481,0.0826)	<0.001	0.03117 (0.0133,0.0496)	<0.001
Total Effect	0.0725 (0.0569,0.0902)	<0.001	0.03767 (0.0212,0.0533)	<0.001
Proportion mediated (control)	0.105 (0.0534,0.1843)	<0.001	0.16971 (0.0640,0.3909)	<0.001
Proportion mediated (treated)	0.1619 (0.0877,0.2620)	<0.001	0.20812 (0.0803,0.4256)	<0.001
ACME (Average)	0.00978 (0.00514,0.01537)	<0.001	0.00720 (0.00299,0.01220)	<0.001
ADE (Average)	0.06270 (0.0462,0.0805)	<0.001	0.0305 (0.0129,0.0488)	<0.001
Proportion mediated	0.13348 (0.0715,0.2228)	<0.001	0.1889 (0.0704,0.4096)	<0.001

### 3.4 Subgroup analysis based on PHQ-9 item scores

We further analyzed the relationship between PHQ-9 item scores, sleep disorders, and gallstones. The results indicated that sleep disorders were significantly associated with higher odds of experiencing depressive symptoms across various PHQ-9 items (OR range: 2.28 to 4.69, all P < 0.05), and that higher PHQ-9 item scores were significantly associated with an increased likelihood of gallstones (OR range: 1.23 to 1.60, all P < 0.05) (**Table 5**). The causal mediation analysis for sleep disorders and depressive symptoms with respect to gallstones, based on individual PHQ-9 items, is presented in **Table 6**. Causal mediation effects were observed for all items except for items 1, 2, and 9. Among these, item 3 had the highest average causal mediation effect (ACME) of 0.0091, with a proportion of mediated effect of 0.2424 (95% CI, 0.0874-0.4950; P < 0.001). Items 4 and 7 also showed substantial mediation effects, with ACME values of 0.0055 and 0.00513, and proportions of mediated effect of 0.1458 (95% CI, 0.0429-0.3257; P = 0.006) and 0.13665 (95% CI, 0.0473-0.2880; P = 0.002), respectively.

**Table 5:** Association between each item of the PHQ score and sleep disorders and gallstone among adults over 20 years of age in 2017–2020 NHANES.

Sleep disorders to depressive symptoms		
Variables	Sleep disorders	
Outcomes	Model 1 OR (95% CI)	Model 2 OR (95% CI)
PHQ-item-1	2.85 (2.57,3.17)	2.79 (2.48,3.15)
PHQ-item-2	3.03 (2.72,3.37)	3.17 (2.81,3.58)
PHQ-item-3	4.82 (4.35,5.35)	4.69 (4.19,5.27)
PHQ-item-4	3.09 (2.79,3.42)	2.92 (2.61,3.28)
PHQ-item-5	2.46 (2.21,2.74)	2.28 (2.03,2.57)
PHQ-item-6	2.68 (2.37,3.03)	2.79 (2.40,3.16)
PHQ-item-7	2.84 (2.52,3.21)	2.89 (2.52,3.31)
PHQ-item-8	2.82 (2.43,3.26)	2.79 (2.37,3.29)
PHQ-item-9	2.99 (2.37,3.78)	3.07 (2.36,4.00)
Depressive symptoms to gallstone		
Outcomes	gallstone	
Variables		
	Model 1 OR (95% CI)	Model 2 OR (95% CI)
PHQ-item-1	1.41 (1.21,1.65)	1.23 (1.03,1.47)
PHQ-item-2	1.36 (1.16,1.59)	1.27 (1.07,1.52)
PHQ-item-3	1.62 (1.40,1.87)	1.47 (1.25,1.73)
PHQ-item-4	1.75 (1.51,2.02)	1.40 (1.19,1.65)
PHQ-item-5	1.69 (1.45,1.97)	1.37 (1.15,1.63)
PHQ-item-6	1.48 (1.24,1.76)	1.41 (1.15,1.73)
PHQ-item-7	1.53 (1.29,1.82)	1.53 (1.26,1.86)
PHQ-item-8	1.58 (1.28,1.93)	1.59 (1.25,2.00)
PHQ-item-9	1.59 (1.12,2.13)	1.60 (1.08,2.30)

**Table 6:** Causal mediation analysis of each PHQ item between sleep disorders and gallstone among adults over 20 years of age in 2017–2020 NHANES.

PHQ-9 items	ACME (Average)		ADE (Average)		Total Effect		Proportion mediated	
	Estimate (95%CI)	P	Estimate (95%CI)	P	Estimate (95%CI)	P	Estimate (95%CI)	P
1	0.00207 (-0.00129,0.00554)	0.23	0.0355 (0.0194,0.0517)	<0.001	0.03756 (0.0220,0.0539)	<0.001	0.05525 (-0.0375,0.1644)	0.23
2	0.00282 (-0.00100,0.00712)	0.15	0.03461 (0.0183,0.0519)	<0.001	0.03744 (0.0213,0.0545)	<0.001	0.0740 (-0.0286,0.2141)	0.15
3	0.0091 (0.00306,0.01494)	0.002	0.02840 (0.0115,0.0461)	<0.001	0.03749 (0.0212,0.0536)	<0.001	0.2424 (0.0874,0.4950)	<0.001
4	0.0055 (0.0167,0.00963)	0.006	0.03232 (0.0149,0.0503)	<0.001	0.03783 (0.0214,0.0548)	<0.001	0.1458 (0.0429,0.3257)	0.006
5	0.00387 (0.000904,0.007403)	0.008	0.03370 (0.0161,0.0497)	<0.001	0.03757 (0.0210,0.0538)	<0.001	0.01025 (0.023,0.231)	<0.001
6	0.00380 (0.000931,0.006961)	0.014	0.03359 (0.0169,0.000747)	<0.001	0.0374 (0.0202,0.0542)	<0.001	0.1023 (0.0247,0.2192)	0.014
7	0.00513 (0.00185,0.00874)	0.002	0.03237 (0.0157,0.0493)	<0.001	0.0375 (0.0211,0.0535)	<0.001	0.13665 (0.0473,0.2880)	0.002
8	0.00370 (0.00116,0.00664)	0.002	0.034 (0.0187,0.0516)	<0.001	0.0377 (0.0225,0.0554)	<0.001	0.09636 (0.0297,0.2215)	0.002
9	0.001686 (-0.000105,0.00389)	0.072	0.03621 (0.0205,0.0536)	<0.001	0.0379 (0.022,0.055)	<0.001	0.0426 (-0.00325,0.12032)	0.072

### 3.5 Subgroup analysis based on age, gender, and obesity

We conducted subgroup analyses based on different age groups, gender, and obesity status to explore the effects of these factors on gallstone risk.

In the age-based subgroups, before adjusting for confounding factors, sleep disorders and depressive symptoms were associated with a higher incidence of gallstones across all age groups, and sleep disorders were also associated with a higher incidence of depressive symptoms (see **Supplementary Tables 2**-**4**). However, after adjusting for confounding factors, significant associations between sleep disorders and depressive symptoms with gallstone incidence remained only in the 40-59 years age group, and the association between sleep disorders and depressive symptoms persisted. Causal mediation analysis for the 40-59 years age group showed a total effect of 0.08499 (95% CI, 0.0828-0.0873; P < 0.001), an average causal mediation effect (ACME) of 0.00894 (95% CI, 0.00860-0.00931; P < 0.001), and a proportion of mediated effect of 0.1052 (95% CI, 0.101-0.109; P < 0.001) (see **Supplementary Tables 5**).

In the gender-based subgroups, both sleep disorders and depressive symptoms were associated with a higher incidence of gallstones, and sleep disorders were also related to a higher incidence of depressive symptoms (see **Supplementary Table 6**-**7**). Causal mediation analysis results showed that, among men, the total effect was 0.02908 (95% CI, 0.00825-0.05115; P = 0.002), with an ACME of 0.00792 (95% CI, 0.00288-0.01430; P < 0.001) and a proportion of mediated effect of 0.2692 (95% CI, 0.0914-0.9566; P < 0.001) (see **Supplementary Table 8**). Among women, the total effect was 0.04849 (95% CI, 0.0223-0.0742; P ≤ 0.001), with an ACME of 0.00589 (95% CI, -0.000368-0.01287; P = 0.068) and a proportion of mediated effect of 0.1191 (95% CI, -0.00877-0.35206; P = 0.068) (see **Supplementary Table 9**).

In the subgroups based on obesity status, both sleep disorders and depressive symptoms were associated with a higher incidence of gallstones, and sleep disorders were also related to a higher incidence of depressive symptoms (see **Supplementary Tables 10**, **11**). Causal mediation analysis results indicated that, among obese individuals, the total effect was 0.04814 (95% CI, 0.0232-0.0765; P ≤ 0.001), with an ACME of 0.00785 (95% CI, 0.000983-0.01636; P = 0.020) and a proportion of mediated effect of 0.1610 (95% CI, 0.018-0.443; P = 0.020) (see **Supplementary Table 12**). Among non-obese individuals, the total effect was 0.03623 (95% CI, 0.0170-0.0558; P ≤ 0.001), with an ACME of 0.00683 (95% CI, 0.00202-0.01262; P = 0.004) and a proportion of mediated effect of 0.1820 (95% CI, 0.0527-0.4548; P = 0.004) (see **Supplementary Table 13**).

## 4 Discussion

This study, based on the 2017-2020 National Health and Nutrition Examination Survey data, explored the relationship between sleep disorders, depressive symptoms, and gallstones. Our findings indicate a significant positive correlation between sleep disorders and the incidence of gallstones. After adjusting for potential confounders (such as age, sex, BMI, alcohol, and physical activity), the risk of gallstones significantly increased among individuals with sleep disorders (OR = 1.49; 95% CI, 1.26-1.76, p < 0.05). Moreover, depressive symptoms partially mediated the association between sleep disorders and gallstones, with approximately 18.89% of the association being mediated by depressive symptoms.

We observed the mediating effect of depressive symptoms in various subgroups, including those aged 40-59, males, and both obese and non-obese groups. However, this mediating effect was not significant among females, which might be due to the potential regulatory role of estrogen in cholesterol metabolism and bile composition.

Sleep disorders can be categorized into several types, such as insomnia disorders, sleep-related breathing disorders, circadian rhythm sleep-wake disorders, hypersomnolence disorders, parasomnias, sleep-related movement disorders, and isolated sleep symptoms (26). However, our study, based on the NHANES sleep disorder questionnaire, could not differentiate between these specific types of sleep disorders, which is a limitation. Despite this, the questionnaire provided a preliminary assessment of generalized sleep disorders. Our results confirm that sleep disorders are an independent risk factor for gallstones, with a significant increase in risk among individuals with sleep disorders (OR = 1.49; 95% CI, 1.26-1.76, p < 0.05). Existing preprint studies indicate a significant association between sleep disorders and the risk of gallstones. These studies show that participants with sleep difficulties were found to have a significantly higher risk of developing gallstones, with adjusted odds ratios ranging from 1.49 to 1.51 (27, 28). However, their study did not explore the potential mediating role of depressive symptoms in the relationship between sleep disorders and gallstones.

Sleep disorders may accelerate gallstone formation through metabolic disturbances, neuroendocrine dysregulation, inflammatory responses, and psychosocial and lifestyle factors. Specifically, sleep disorders, particularly OSAHS, are closely associated with metabolic disturbances, including insulin resistance and metabolic syndrome, which are significant risk factors for gallstones (29, 30). Sleep disorders reduce insulin sensitivity, perturbing bile composition and promoting cholesterol supersaturation in bile, which are key steps in gallstone pathogenesis (2). Additionally, sleep disorders may activate the hypothalamic-pituitary-adrenal (HPA) axis, increasing cortisol secretion and affecting bile metabolism and cholesterol concentration (31). Excess cholesterol secretion is a major cause of cholesterol stone formation, and high cortisol levels may also impact gallbladder contraction, leading to bile stasis and an increased risk of gallstone formation (32). Furthermore, sleep disorders may exacerbate chronic inflammation in the gallbladder, with elevated inflammatory markers such as interleukin-6 (IL-6) and C-reactive protein (CRP) being associated with increased gallstone risk (33, 34). Sleep disorders are often linked with poor lifestyle choices and psychological stress, which may further contribute to gallstone formation by altering dietary habits and increasing smoking and alcohol consumption.

Therefore, we propose a hypothesis: Social psychological factors may act as mediators in the relationship between sleep disorders and gallstones, indirectly affecting the occurrence of gallstones by influencing sleep disorders. Our study further validates that depressive symptoms may play a mediating role between sleep disorders and gallstones. After adjusting for confounders, depressive symptoms significantly increased the risk of gallstones (OR = 1.77; 95% CI, 1.39-2.24, p < 0.05) and enhanced the impact of sleep disorders on gallstone risk. This finding is consistent with previous research results (35–37). Mediation analysis indicates that 18.89% of the association between sleep disorders and gallstones seems to be mediated by depressive symptoms. Subgroup analyses reveal differences in the mediating effect of various PHQ-9 items on sleep disorders and gallstones. Among different age groups, depressive symptoms mediate the relationship between sleep disorders and gallstones primarily in the 40-59 age group, while no significant associations were observed in the 20-39 and ≥60 age groups. Similarly, mediation effects are observed in males, obese, and non-obese groups. These results suggest that improving depressive symptoms in specific populations may reduce the risk of gallstones associated with sleep disorders. The lack of significance in females might be attributed to estrogen’s role in regulating cholesterol metabolism and bile composition, which may reduce the mediating effect of depressive symptoms.

This study is the first to discuss the impact of sleep disorders on gallstones and to reveal the potential role of depressive symptoms in gallstone formation. Clinical practice should incorporate the identification and management of sleep disorders and depressive symptoms as crucial components of gallstones prevention. Early intervention for high-risk groups, particularly those over 40 years old and males, may reduce gallstone incidence. Additionally, our results suggest that clinical attention should extend beyond traditional gallstone risk factors (such as obesity and diet) to include patient sleep and mental health.

Despite providing robust evidence on the association between sleep disorders, depressive symptoms, and gallstones, this study has limitations. Firstly, the cross-sectional nature of the study limits causal inference. Future research should employ longitudinal designs to better determine the temporal sequence and causal pathways between sleep disorders, depressive symptoms, and gallstones. Secondly, self-reported data on sleep disorders and depressive symptoms may be subject to recall and subjective biases. Future studies could use more objective assessment tools, such as polysomnography (PSG) and biomarkers (e.g., cortisol and bile components), to further validate these results. Although we controlled for various potential confounders (e.g., BMI, diet, physical activity), other unconsidered confounders may impact the results, such as genetic susceptibility, long-term medication use (e.g., ursodeoxycholic acid and statins), and other chronic diseases (e.g., non-alcoholic fatty liver disease (NAFLD), chronic hemolytic diseases). Future research should further account for these factors. Finally, sleep disorder data from the questionnaire did not allow for specific classification of sleep disorder types. Future research should consider categorizing different types of sleep disorders for more precise results.

## 5 Conclusion

In summary, depressive symptoms and sleep disorders are independently associated with gallstones, and sleep disorders are also associated with a higher risk of depressive symptoms. Depressive symptoms may mediate the relationship between sleep disorders and gallstones. Mediating effects of depressive symptoms on sleep disorders and gallstones were observed in the 40-59 age group, males, and both obese and non-obese populations. Early prevention, detection, and treatment are needed for specific populations. Improving depressive symptoms may reduce the likelihood of gallstones caused by sleep disorders.

## Data Availability

Publicly available datasets were analyzed in this study. This data can be found here: https://www.cdc.gov/nchs/nhanes/index.htm.
