# Supplementary material for: Depressive symptoms mediate the association between sleep disorders and gallstone disease: a causal mediation analysis of NHANES 2017–2020
Source: Front Psychiatry. 2025 Apr 17;16:1434889. doi: 10.3389/fpsyt.2025.1434889 (PMC12043569; doi:10.3389/fpsyt.2025.1434889)
Supplement: Supplementary file 1 [file DataSheet1.pdf]

Supplementary Table 1: Association between depressive symptoms, sleep disorders, and gallstone in populations without sleep-related medication among adults over 20 years of age in 2017-2020 NHANES.

Outcomes	Gallstone<br>OR (95%CI)<br>P value		Depressive symptoms<br>OR(95%CI)<br>P value	
Variables	Model 1	Model 2	Model 1	Model 2
Sleep disorders	1.94(1.67,2.25)<br><0.001	1.48(1.25,1.75)<br><0.001	5.37(4.56,6.33)<br><0.001	5.41(4.49,6.52)<br><0.001
Depressive symptoms	2.07(1.68,2.54)<br><0.001	1.81(1.42,2.29)<br>0.030	-	-

Supplementary Table 2: Association between depressive symptoms, sleep disorders, and gallstone in populations among adults aged 20-39 years in 2017-2020 NHANES.

Outcomes	Gallstone<br>OR (95%CI)<br>P value		Depressive symptoms<br>OR(95%CI)<br>P value	
Variables	Model 1	Model 2	Model 1	Model 2
Sleep disorders	1.99(1.34,2.91)<br><0.001	1.06(0.66,1.68)<br>0.794	5.86(4.39,7.83)<br><0.001	5.13(3.70,7.14)<br><0.001
Depressive symptoms	1.72(0.99,2.83)<br>0.040	0.91(0.46,1.58)<br>0.771	-	-

Supplementary Table 3: Association between depressive symptoms, sleep disorders, and gallstone in populations among adults aged 40-59 years in 2017-2020 NHANES.

Outcomes	Gallstone<br>OR (95%CI)<br>P value		Depressive symptoms<br>OR(95%CI)<br>P value	
Variables	Model 1	Model 2	Model 1	Model 2
Sleep disorders	2.25(1.74,2.89)<br><0.001	2.03(1.53,2.70)<br><0.001	7.47(5.57,10.14)<br><0.001	6.82(4.95,9.54)<br><0.001
Depressive symptoms	2.97(2.14,4.10)<br><0.001	2.47(1.71,3.53)<br><0.001	-	-

Supplementary Table 4: Association between depressive symptoms, sleep disorders, and gallstone in populations among adults over 60 years in 2017-2020 NHANES.

Outcomes	Gallstone<br>OR (95%CI)<br>P value		Depressive symptoms<br>OR(95%CI)<br>P value	
Variables	Model 1	Model 2	Model 1	Model 2
Sleep disorders	1.54(1.25,1.90)<br><0.001	1.30(1.03,1.64)<br>0.026	4.47(3.42,5.89)<br><0.001	4.15(3.04,5.72)<br><0.001
Depressive	1.75(1.27,2.37)	1.42(0.97,2.04)	-	-

symptoms	<0.001	0.066

Supplementary Table 5: Causal mediation analysis of depressive symptoms in the association between sleep disorders and gallstones among adults aged 40-59 years in 2017-2020 NHANES.

Type	Estimate (95 % CI)	P value	Estimate (95 % CI)	P value
	Model 1		Model 2	
ACME (control)	0.00789 (0.00774,0.00806)	<0.001	0.00644 (0.00618,0.00672)	<0.001
ACME (treated)	0.01547 (0.0152,0.0158)	<0.001	0.01145 (0.0110,0.0119)	<0.001
ADE (control)	0.08633 (0.0851,0.0876)	<0.001	0.07354 (0.0716,0.0756)	<0.001
ADE (treated)	0.09391 (0.0926,0.0952)	<0.001	0.07885 (0.0764,0.0807)	<0.001
Total Effect	0.10181 (0.100,0.103)	<0.001	0.08499 (0.0828,0.0873)	<0.001
Proportion mediated (control)	0.07750 (0.0759,0.0793)	<0.001	0.07573 (0.0726,0.0790)	<0.001
Proportion mediated (treated)	0.15199 (0.149,0.155)	<0.001	0.13467 (0.13007,0.14133)	<0.001
ACME (Average)	0.01168 (0.0115,0.0119)	<0.001	0.00894 (0.00860,0.00931)	<0.001
ADE (Average)	0.09012 (0.0889,0.0914)	<0.001	0.07604 (0.0740,0.0781)	<0.001
Proportion mediated	0.11475 (0.1127,0.01169)	<0.001	0.1052 (0.101,0.109)	<0.001

Supplementary Table 6: Association between depressive symptoms, sleep disorders, and gallstone in populations among male adults over 20 years of age in 2017-2020 NHANES.

Outcomes	Gallstone OR (95%CI) P value		Depressive symptoms OR(95%CI) P value	
Variables	Model 1	Model 2	Model 1	Model 2
Sleep disorders	1.88(1.43,2.47) <0.001	1.58(1.16,2.14) 0.003	5.72(4.46,7.32) <0.001	5.61(4.21,7.53) <0.001
Depressive symptoms	2.39(1.61,3.46) <0.001	2.56(1.62,3.94) <0.001	-	-

Supplementary Table 7: Association between depressive symptoms, sleep disorders, and gallstone in populations among female adults over 20 years of age in 2017-2020

NHANES.

Outcomes	Gallstone OR (95%CI) P value		Depressive symptoms OR(95%CI) P value	
Variables	Model 1	Model 2	Model 1	Model 2
Sleep disorders	1.90(1.59,2.27) <0.001	1.47(1.20,1.80) <0.001	5.33(4.32,6.60) <0.001	5.10(4.01,6.51) <0.001
Depressive symptoms	1.77(1.38,2.24) <0.001	1.49(1.12,1.97) 0.005	-	-

Supplementary Table 8: Causal mediation analysis of depressive symptoms in the association between sleep disorders and gallstones among male adults over 20 years of age in 2017-2020 NHANES.

Type	Estimate (95 % CI)	P value	Estimate (95 % CI)	P value
	Model 1		Model 2	
ACME (control)	0.00590 (0.0018,0.0121)	0.004	0.00699 (0.00241,0.01301)	<0.001
ACME (treated)	0.00889 (0.0030,0.0170)	0.004	0.00885 (0.0034,0.0158)	<0.001
ADE (control)	0.03217 (0.0123,0.0514)	<0.001	0.02023 (2.54e-05,0.0406)	0.05
ADE (treated)	0.03516 (0.0143,0.0555)	<0.001	0.0221 (2.81e-05,0.0436)	0.05
Total Effect	0.0411 (0.0214,0.0611)	<0.001	0.02908 (0.00825,0.05115)	0.002
Proportion mediated (control)	0.1373 (0.0378,0.3685)	0.004	0.2346 (0.0747,0.9543)	0.002
Proportion mediated (treated)	0.2120 (0.0685,0.4652)	0.004	0.3039 (0.110,0.959)	0.002
ACME (Average)	0.00739 (0.00239,0.01426)	0.004	0.00792 (0.00288,0.01430)	<0.001
ADE (Average)	0.0337 (0.0132,0.0531)	<0.001	0.02116 (2.68e-05,0.0420)	0.05
Proportion mediated	0.1746 (0.0541,0.4147)	0.004	0.2692 (0.0914,0.9566)	<0.001

Supplementary Table 9: Causal mediation analysis of depressive symptoms in the association between sleep disorders and gallstones among female adults over 20 years of age in 2017-2020 NHANES.

Type	Estimate (95 % CI)	P value	Estimate (95 % CI)	P value
	Model 1		Model 2	

ACME (control)	0.00753 (0.00164,0.01419)	0.008	0.005347 (-0.000318,0.01192)	0.068
ACME (treated)	0.01078 (0.00238,0.01968)	0.008	0.00643 (-0.000399,0.01370)	0.068
ADE (control)	0.0766 (0.0507,0.1023)	<0.001	0.04206 (0.0158,0.0680)	0.002
ADE (treated)	0.0798 (0.0538,0.1060)	<0.001	0.04315 (0.0162,0.0690)	0.002
Total Effect	0.0874 (0.0627,0.1132)	<0.001	0.04849 (0.0223,0.0742)	<0.001
Proportion mediated (control)	0.0839 (0.019,0.179)	0.008	0.1066 (-0.007789,0.3327)	0.068
Proportion mediated (treated)	0.1232 (0.0294,0.2383)	0.008	0.1317 (-0.009751,0.3680)	0.068
ACME (Average)	0.00916 (0.00202,0.01695)	0.008	0.00589 (-0.000368 ,0.01287)	0.068
ADE (Average)	0.0782 (0.0522,0.1042)	<0.001	0.04261 (0.0160,0.0684)	0.002
Proportion mediated	0.1036 (0.0241,0.2117)	0.008	0.1191 (-0.00877,0.35206)	0.068

Supplementary Table 10: Association between depressive symptoms, sleep disorders, and gallstone in populations among obesity adults over 20 years of age in 2017-2020 NHANES.

Outcomes	Gallstone OR (95%CI) P value		Depressive symptoms OR(95%CI) P value	
Variables	Model 1	Model 2	Model 1	Model 2
Sleep disorders	1.68(1.39,2.03) <0.001	1.47(1.19,1.82) <0.001	5.27(4.18,6.68) <0.001	5.14(3.97,6.70) <0.001
Depressive symptoms	1.74(1.33,2.25) <0.001	1.60(1.19,2.15) 0.002	-	-

Supplementary Table 11: Association between depressive symptoms, sleep disorders, and gallstone in populations among non-obesity adults over 20 years of age in 2017-2020 NHANES.

Outcomes	Gallstone OR (95%CI) P value		Depressive symptoms OR(95%CI) P value	
Variables	Model 1	Model 2	Model 1	Model 2
Sleep disorders	2.12(1.67,2.69) <0.001	1.65(1.27,2.15) <0.001	5.52(4.37,6.99) <0.001	5.50(4.24,7.16) <0.001

Depressive symptoms	2.11(1.47,2.95) <0.001	2.15(1.44,3.15) <0.001	-	-

Supplementary Table 12: Causal mediation analysis of depressive symptoms in the association between sleep disorders and gallstones among obesity adults over 20 years of age in 2017-2020 NHANES.

Type	Estimate (95 % CI)	P value	Estimate (95 % CI)	P value
	Model 1		Model 2	
ACME (control)	0.00844 (0.00251,0.01575)	0.004	0.00719 (0.000837,0.015369)	0.020
ACME (treated)	0.01114 (0.00342,0.02010)	0.004	0.00851 (0.00111,0.01741)	0.020
ADE (control)	0.05847 (0.0317,0.0865)	<0.001	0.03963 (0.0359,0.0378)	0.002
ADE (treated)	0.06117 (0.0340,0.0891)	<0.001	0.04094 (0.0142,0.0701)	0.002
Total Effect	0.06961 (0.0438,0.0966)	<0.001	0.04814 (0.0232,0.0765)	<0.001
Proportion mediated (control)	0.11849 (0.0329,0.2702)	0.004	0.14674 (0.0165,0.4254)	0.020
Proportion mediated (treated)	0.15798 (0.0493,0.3207)	0.004	0.17527 (0.0196,0.4255)	0.020
ACME (Average)	0.00979 (0.00297,0.01789)	0.004	0.00785 (0.000983,0.01636)	0.020
ADE (Average)	0.05982 (0.0330,0.0879)	<0.001	0.04029 (0.0146,0.0708)	0.002
Proportion mediated	0.13823 (0.0414,0.2954)	0.004	0.16100 (0.018,0.443)	0.020

Supplementary Table 13: Causal mediation analysis of depressive symptoms in the association between sleep disorders and gallstones among non-obesity adults over 20 years of age in 2017-2020 NHANES.

Type	Estimate (95 % CI)	P value	Estimate (95 % CI)	P value
	Model 1		Model 2	
ACME (control)	0.00468 (0.000795,0.009514)	0.004	0.00586 (0.00164,0.01147)	0.004
ACME (treated)	0.00789 (0.00156,0.01543)	0.004	0.00779 (0.00229,0.0489)	0.004
ADE (control)	0.04921 (0.0298,0.0693)	<0.001	0.02844 (0.0097,0.0378)	0.006
ADE (treated)	0.05242 (0.0323,0.0726)	<0.001	0.03037 (0.0103,0.0515)	0.006

Total Effect	0.05710 (0.0372,0.0766)	<0.001	0.03623 (0.0170,0.0558)	<0.001
Proportion mediated (control)	0.07863 (0.014,0.182)	0.004	0.1541 (0.0396,0.4252)	0.004
Proportion mediated (treated)	0.13817 (0.0254,0.2720)	0.004	0.2099 (0.064,0.486)	0.004
ACME (Average)	0.00629 (0.00117,0.01242)	0.004	0.00683 (0.00202,0.01262)	0.004
ADE (Average)	0.0508 (0.0311,0.0708)	<0.001	0.02940 (0.0101,0.0497)	0.006
Proportion mediated	0.1084 (0.0196,0.2251)	0.004	0.18202 (0.0527,0.4548)	0.004
